# Comparison of Happiness and Spiritual Well-Being among the Community Dwelling Elderly and those who Lived in Sanitariums

**Published:** 2015-07

**Authors:** Mohsen Adib-Hajbaghery, Mona Faraji

**Affiliations:** Department of Medical Surgical Nursing, Kashan University of Medical Sciences, Kashan, Iran

**Keywords:** Aged, Community, Happiness, Hospices, Spirituality

## Abstract

**Background:**

Several studies are available on the lifestyle, psychological and mental health of the elderly adults. This study aimed to compare the spiritual well-being and happiness in the elderly who lived in sanitariums with those lived in the community.

**Methods:**

A comparative study was conducted on 384 elderly adults. A census sampling was used in sanitariums and a convenience sampling was performed to select the community dwelling (CD) older adults. A demographic questionnaire, the Pauloutzian and Ellison’s spiritual well-being scale and the Oxford happiness questionnaire were used in this study. Descriptive statistics and Kolmogorov-Smirnov, Chi-square and Mann-Whitney U tests and Spearman correlation coefficient were employed for data analysis, using the SPSS software, version 13.0.

**Results:**

From the total participants, 56% were CD elderly and 44% were in sanitariums. Among the CD older adults, no one was at a high level of spiritual well-being while in sanitariums 24.4% were at a high level of spiritual well-being. Also, 71.2% of the community dwelling older adults were at a high level of happiness while only 3.6% of those living in sanitariums expressed a high level of happiness. A significant association was found between the level of spiritual well-being and happiness in those who lived in sanitariums (r=0.177, P<0.021).

**Conclusion:**

Most of the elderly living in the community and in sanitariums showed moderate spiritual well-being and low happiness. Therefore, nurses and health authorities are responsible not only to inform the community about the importance of spiritual well-being and happiness, but also to establish some strategies in this regard.

## Introduction

By getting closer to the third millennium, aging seems to be a major global fact. In 2011, about 800 million people were 60 years old or more, but up to 2025 these statistics would be 1.2, and up to 2050 it will be about 2 billion people.^1^ About two-thirds of the elderly people are living in developing countries, and up to 2025 these statistics will be about 75%.^1^ By increasing the number of the elderly population, their health issues will be more challenging. Reduction of social communication, loss of relatives, and physical and mental illnesses among the aged people lead to a big challenge to reserve healthy lifestyle.^2^

In Iran, the proportion of the older adults has increased from 7.22% of the total population in 2006 to 8.20% in 2011.^3^ As a result, some appropriate, multidimensional, economical and socio-cultural activities in public and state organizations should be arranged. In literature, there is a distinction between religion and spirituality.^4^^,^^5^A vast majority of written literature have been devoted to the relationship between healthiness and religious-spiritual state.^6^^,^^7^Beliefs, manners and some rituals which are used as a tradition are regarded as religion while spirituality is regarded as a personal effort to understand the life meaning and goals.^7^ There is an association between improvement in the individual’s spiritual aspects and reduction in the rates of depression and suicide.^8^^,^^9^Recently, spirituality and its elements have considerably been noticed in nurse-patient relationships.^10-13^Increases in self-control, self-esteem and trust is among the benefits of spirituality, as mentioned by some authors.^14^ However, ignoring the patients’ spiritual aspects may result in undesirable consequences in the process of treatment. It is reported that provision of patients’ spiritual needs could not only reduce the negative effects of stress and fear but also would decrease the mortality rates.^12^^,^^15^^,^^16^ Moreover, associations were observed between spirituality and mental health,^17^self-esteem, social skills,^18^ and quality of life (QOL) of healthy adults,^19^ and patients with chronic heart failure.^20^

Spiritual well-being is one of the most important aspects of human health.^21^ The perception of spiritual well-being may be examined from two different points of religious well-being and existential one. The religious well-being includes individual’s perception of health in his/her spiritual life related to a superior power. The existential well-being considers the individual’s social and mental concerns and the person’s ways of coping with the self, society and environment.^22^ In recent years, nursing theorists have paid close attention to the spiritual powers as a source of happiness, peacefulness and energy in older adults.^23^

In a study of patients’ attitudes towards the role of spirituality in healing, that was conducted in Tehran, it was reported that there was a significant relationship between patients’ spiritual beliefs and their recovery and QOL.^24^In another study in the Eastern Azerbaijan, spirituality was cited as one of the key features affecting the elderly peoples’ lifestyle.^2^ A recent study in Tehran has also compared the spiritual health of older adults living in sanitariums with those living at their home. Interestingly, the mean score of spiritual health was significantly higher in those living in sanitariums.^25^However, in another study it has been reported that the mental health and emotional well-being were significantly lower in older adults living in sanitariums when compared with those living in their homes.^26^

As a public approach, happiness is meant to exist in a state of joyfulness.^27^ Studies showed that happy people are more successful in different aspects of life, such as marriage, friendship, income, job and health.^28^ In a recent study in Tehran, that was conducted on 389 aged adults living with their families, a direct significant relationship was found between religious orientation and happiness.^29^ It has also been reported that the level of happiness is significantly lower among the older adults living in sanitariums than community dwelling older adults.^30^ A study in Shahroud, Iran, has also reported that the rate of depression and cognitive disorders was significantly higher among the aged people living in a sanitarium than those who lived at their home.^31^

Several studies are available on the lifestyle.^2^ mental health,^26^ depressions, spiritual and religious beliefs^5^ of the elderly adults. Also, some studies have investigated the relationship between spirituality and mental health^8^^,^^9^in aged people. However, still there are questions about the state of the spiritual well-being and happiness among the community dwelling older adults and those living in sanitariums, and if there is any correlation between spiritual well-being and happiness in older adults. Therefore, this study aimed to assess and compare the levels of spiritual well-being and happiness among the community dwelling elderly and those who lived in sanitariums.

## Materials and Methods

A comparative study was conducted on 384 elderly adults in Arak and Kashan cities from January to March 2014. A total of 108 community dwelling elderly in each city and 81 and 87 elderly people who lived in the sanitariums in Arak and Kashan participated in this study. Inclusion criteria were the age of 60 years or over, ability to speak in Persian language, having no hearing and communication problems, and willing to participate in the study. A participant’s decision to withdraw from the study while completing the questionnaires and incomplete answering the questionnaires were selected as exclusion criteria. 

Sample size was calculated using the Cochrane formula for estimating the maximum sample needed. Using a type one error of 0.05, a probability value of 0.5 and the sampling error of 0.05, we estimated a sample of 384 subjects to be used for the study. 

To recruit the elderly living in sanitariums, a census sampling method was used to select all the participants with inclusion criteria in the two sanitariums in Kashan and Arak cities. However, a convenience sampling was performed to select the community dwelling older adults. To this end, two valleys, two banks, two parks and two main squares were randomly selected in each city. Then, all the houses in the selected valleys were referred to and the elderly residents with inclusion criteria were recruited if they agreed to take part in the study after a brief explanation of the study objectives. Moreover, the researchers performed regular daily visits of the selected banks and parks to select the elderly adults with inclusion criteria among those who referred to these sites. This part of sampling continued till the sample size was completed. 

A three part questionnaire was used in this study. The first part consisted of 10 questions on age, gender, education level, residential place, people living with the elderly, job, monthly income, walking ability, having any chronic disease, and history of smoking. The Pauloutzian and Ellison’s spiritual well-being scale (SWBS) and the Oxford happiness questionnaire (OHQ) were used as the second and the third parts of the instruments. 

The SWBS scale is consisted of 20 items, 10 related to religious health and 10 about the existential health. All items were answered on a 6 point Likert scale ranging from strongly disagree (=1) to strongly agree (=6). The scoring was reversed in negative items. The sum of scores ranged from 20 to 120 and a higher score indicated the higher level of spiritual well-being. Then, the scores from 20-40, 41-99, and 100-120 were regarded as low, moderate and high levels of spiritual well-being, respectively. The SWBS scale had been previously translated into Persian language and showed appropriate content validity and internal consistency (α=0.92).^21^

The OHQ questionnaire comprised 29 items, each involving the selection of one of four options that were different for each item. All items were scored on a 4-point Likert scale ranging from zero to 3. The highest score of this questionnaire was 87, and the lowest 0. Then, scores from 40 to 42 could be regarded as normal. Obtaining scores higher than 42 reflected higher levels of happiness. As a result, the higher the score, the higher the level of happiness. The OHQ questionnaire was previously translated into Persian language and showed appropriate content validity and internal consistency (α=0.87).^32^

Because most of the participants were illiterate, the instrument was completed through interviews. The questions were asked by the second researcher in a simple and clear way and their answers were entered into the study instrument. 

*Ethical Consideration*

The research objectives were explained to all participants. All of them signed a written informed consent to participate and were assured about the confidentiality of their personal information. Ethical issues of the study were approved in the research ethics committee in Kashan University of Medical Sciences. Also, permissions were obtained from sanitarium managers. 

*Data Analysis*

Statistical analysis was conducted using SPSS software, version 13.0. Descriptive statistics (frequency, percentage, mean and standard deviation) were calculated. Inferential statistics were also used. The Kolmogorov-Smirnov Test revealed that the distribution of the variables was not normal. Then, Chi-square-test was used to compare the two groups in terms of their demographics. Mann-Whitney U test was used to compare the mean scores of the two groups. Also, Chi-square-test was used to test the relationship between happiness and also spiritual well-being in sanitarium and community dwelling older adults. Spearman correlation coefficient was used to examine the association between spiritual well-being and happiness scores. 

## Results

From a total of 384 participants, 215 (56%) were community dwelling elderly and 169 (44%) were in sanitariums. Totally, 67.7% of the participants (including 59.1% of the participants in community and 78.7% of those living in sanitariums) were males. The mean age of the participants was 73.47±8.93 years (73.05±8.12 years in the community elderly and 73.76±6.53 in those living in the sanitarium, P=0.35). Moreover, no significant differences were observed between the elderly in the community and in sanitarium in terms of education level (P=0.16), having a chronic disease (P=0.69) and walking ability (P=0.068). 

The mean of spiritual well-being score was 54.15±15.87 in the community dwelling elderly adults and 71.65±22.87 of those in the sanitariums (P<0.001). Moreover, the mean of happiness scores was 51.48±13.14 in the community dwelling elderly while it was 24.49±8.42 in those in the sanitariums (P<0.001).

As shown in Table 1, no one among the community dwelling older adults got a high score of spiritual well-being while 24.3% of the elderly living in sanitariums were at a high level of spiritual well-being. The Chi-square test showed a significant relationship between spiritual well-being and residential place (P=0.001). Moreover, the majority of the older adults in sanitariums were in a low level of happiness while most of the community dwelling elderly adults obtained high level scores in happiness. The Chi-square test showed a significant relationship between the levels of happiness and residential place (P=0.001) (Table 1).

**Table 1:** Levels of happiness and spiritual well-being in the elderly living in sanitarium or community

**Residential place**	**Spiritual well-being, No. (%)**			**Chi-square**		**Happiness, No. (%)**			**Chi-square**	
	**Low **	**Moderate **	**High **	**^χ2^**	**P**	**Low **	**Normal **	**High **	**^χ2^**	**P**
Sanitarium	19 (11.2)	109 (64.5)	41 (24.3)	64.83	0.001	157 (92.8)	6 (3.6)	6 (3.6)	196.38	0.001
Community	58 (27)	157 (73)	0 (0)			47 (21.8)	15 (7)	153 (71.2)		
Total	77 (20.1)	266 (69.3)	41 (10.7)			204 (53.1)	21 (5.5)	159 (41.4)		

Significant differences were observed between the mean scores of spiritual well-being of the elderly living in sanitariums and those living in the community in terms of different demographic variables (P<0.001), (Table 2).

**Table 2:** The differences in spiritual well-being scores of the two elderly groups in terms of demographic variables*

**Variables**	**Residential place**		**P**
	**Sanitarium**	**Community**	
Gender			
Male	65.41±21.22	48.17±10.83	0.001
Female	93.83±12.81	62.77±17.91	0.001
P value	0.001	0.001	
Walking ability			
Yes	70.91±20.83	55.96±16.92	0.001
No	74.60±22.61	48.10±10.64	0.001
P value	0.295	0.014	
Having a chronic disease			
Yes	72.21±23.11	53.70±14.90	0.001
No	70.26±23.12	54.8±17.22	0.001
P value	0.381	0.850	
History of smoking			
Yes	63.10±22.14	47.38±10.22	0.001
No	80.12±20.53	56.77±16.81	0.001
P value	0.001	0.001	
Monthly income (equal to USD)			
<100	74.82±21.82	48.87±10.27	0.001
100-600	62.33±33.48	53.77±17.29	0.001
600-1000	-	53.25±11.83	-
No income	71.39±23.43	59.81±16.62	0.001
P value	0.639	0.001	
Education level			
Illiterate	73.04±22.69	58.44±16.28	0.001
Primary school	65.45±22.84	54.76±16.41	0.001
High school	70.62±25.03	45.62±10.01	0.001
P value	0.282	0.001	
Job			
Unemployed	70.25±23.2	-	-
Self-employed	-	51.33±13.7	-
Retired	74.11±22.36	52.8±14.71	0.001
P value	0.514	0.525	

^*^All data presented as Mean±SD

Moreover, significant differences were observed between the mean scores of happiness of the elderly living in sanitariums and those living in the community in terms of different demographic variables (P<0.001), (Table 3).

**Table 3:** The differences in happiness scores of the two elderly groups in terms of demographic variables*

**Variables **	**Residential place**		**P**
	**Sanitarium **	**Community **	
Gender			
Male	23.58±7.56	53.02±13.01	0.001
Female	27.44±10.82	49.26±13.08	0.001
P value	0.228	0.019	
Walking ability			
Yes	24.94±8.51	53.51±11.26	0.001
No	25.96±8.91	50.46±13.89	0.001
P value	0.631	0.289	
Having a chronic disease			
Yes	25.13±8.79	51.61±14.14	0.001
No	23.25±7.85	51.3±11.59	0.001
P value	0.158	0.623	
History of smoking			
Yes	22.63±7.63	45.47±12.45	0.001
No	26.24±8.94	53.81±12.68	0.001
P value	0.083	0.001	
Education level			
Illiterate	25.83±8.68	54.58±12.12	0.001
Primary school	20.06±7.06	47.16±12.72	0.001
High school	21.02±4.31	54.15 ±13.63	0.001
P value	0.016	0.001	
Income			
<100	25.24±8.85	48.92±13.90	
100-600	20.0±0.0	51.66±13.05	
600-1000	-	62.45±7.54	
No income	24.58±9.46	49.14±12.09	
Job	0.245	0.001	
Unemployed	23.72±8.31	-	-
Self-employed	-	57.01±12.71	-
Retired	25.89±8.72	53.23±13.42	0.001
P value	0.246	0.344	

^*^All data presented as Mean±SD

Using the Spearman’s correlation coefficient, we found a significant association between the levels of spiritual well-being and happiness in those who lived in sanitariums (r=0.177, P<0.021). However, no significant association was found between the levels of spiritual well-being and happiness in the community dwelling elderly (r=0.079, P=0.251). 

## Discussion

This study aimed to compare happiness and spiritual well-being among the community dwelling elderly adults and those who lived in sanitariums. To the best of our knowledge, no similar studies are available and this was the first study investigating both variables simultaneously in a sample of aged people. 

The present study showed that the majority of the community dwelling older adults and those who lived in sanitariums were at moderate and low levels of spiritual well-being. The level of spiritual well-being was lower to some extent in the community dwelling older adults as no one in this group got a high score at spiritual well-being. A recent study in Tehran has also examined the spiritual well-being of senior citizens living in sanitarium and home residents, reporting that most of the elderly in both settings had a moderate level of spiritual well-being though the mean spiritual health score was significantly higher among the elderly who lived in sanitarium.^25^ Consistent with the present study, a recent study has investigated the relationship between spiritual well-being and QOL among elderly people residing in Kahrizak sanitarium, indicating that the mean score of spiritual well-being was at a moderate level.^33^The higher level of spiritual well-being among the elderly who lived in sanitariums may be attributed to the fact that they were in continual contact with other aged people like themselves. Therefore, they solved their emotional problems easier than those who were community dwelling.

The present study revealed that the level of happiness was higher among community dwelling older adults than those who lived in sanitariums. This finding was consistent with a previous study which compared the level of happiness among the elderly living at home and the senior home residents.^34^ Another study has also reported that depression was more prevalent among the elderly living in sanitariums than those living at their home.^35^ However, our finding was in contrast with the results of a recent study that investigated the level of happiness among a sample of elderly people in Thailand and reported that the majority of community dwelling elderly adults had a moderate or high level of happiness.^36^Although this conflict may be attributed to the differences in sample size, socio-cultural factors and family structures, the difference in the study instrument might also be a reason since they assessed happiness using a researcher-made questionnaire that might be different from that used in the present study in terms of the basic questions and constructs.

The present study showed that even with respect to demographic variables, the means of happiness scores were significantly higher in the elderly living in the community than those living in sanitariums. However, the means of spiritual health, by all demographic variables, were significantly higher in the elderly who lived in sanitariums than those living in the community. 

In the present study, no significant gender differences were observed in happiness scores of the elderly living in sanitariums. However, in the community dwelling elderly people the mean score of happiness was significantly higher in males than in females. Also, in both settings (i.e. sanitariums and community), the spiritual health of the older females was significantly higher than males. This finding was consistent with two previous studies in Iran^26^ and India.^37^ These findings can be attributed to the fact that the males are usually more active and consider themselves to be more important in achieving the goals of life in their middle-aged and adulthood. However, with the retirement and declining in their roles, their feeling of being important is diminished and consequently would affect their level of spiritual well-being.

The present study showed that in sanitariums, no significant difference was observed in happiness mean scores of smokers and non-smokers. However, in community dwelling older adults, the mean happiness score was significantly higher among non-smokers than in smokers. Moreover, in both settings, the spiritual health of older people who did not smoke was higher than that of smokers. These finding are consistent with the results of a study that investigated the relationship between life satisfaction and health behavior.^38^ Perhaps, indecisiveness and despair after the retirement have led people toward smoking. Then, the lower levels of spiritual well-being in smokers might indirectly be attributed to the state of wondering and having no specific aims –but not to smoking- at these last stages of the life.

Our results showed that, in sanitariums, no significant difference was observed between the spiritual health scores of individuals with and without walking ability. This finding was consistent with the results of a previous study that investigated the factors affecting happiness.^39^ This finding may be attributed to the fact that the elderly living in sanitariums are usually confined in a limited space, regardless of their ability to walk. On the contrary, the community dwelling elderly with ability to walk had higher spiritual wellbeing scores than those unable to walk. However, the ability to walk induced no significant difference in the seniors’ happiness mean scores in both settings. Peoples’ ability to walk is a main factor affecting their social relationships, their feeling of effectiveness, joy and psychological health. Therefore, the insignificant effect between ability to walk and happiness in the present study indicates the need for further study in this area.

In the present study, the illiterate people who lived in sanitariums and the community dwelling elderly people with primary education obtained the highest mean scores of happiness. Therefore, it seems that illiterate or low-literate individuals were happier than those with higher levels of education. Although this finding may be attributed to the number and distribution of the elderly people participating in this study, it might also be attributed to the fact that people with lower levels of education usually have lower levels of expectancies in their lives and then experience lower levels of tension in the life conditions. Other studies reported mixed results regarding the relationship between education and happiness. In some studies, an indirect association was reported^39^ and some others found no relationship between the two variables.^25^ That’s why this issue needs further investigation. In addition, although we found no significant relationship between the education level and the mean of spiritual well-being in the elderly lived in sanitariums, the community dwelling elderly with lower levels of education obtained higher scores of spiritual well-being. Previous studies revealed varying results as to the relationship between education and spiritual well-being. In some studies, a direct association was reported;^40^ some reported that people with low literacy are more religious and perform more spiritual activities^41^and some others found no relationship between the two variables.^25^

In the present study, no significant difference was observed in happiness and spiritual well-being mean scores of the elderly people (either in sanitarium or in the community) in terms of their previous job and having a chronic illness. Moreover, in sanitariums, no significant differences were observed in happiness and well-being mean scores of the elderly people with different levels of income. However, in the community dwelling elderly people the mean happiness score was higher in those with higher income. Studies have shown that the happiness and spiritual well-being of older people are decreased in those with chronic illnesses.^42^^,^^43^ However, a study has reported that a high level of spiritual well-being improves the intensity of a chronic illness in older adults.^44^

In the present study, a direct correlation was observed between the participants’ scores in spiritual well-being and happiness. It means that an increase in happiness would result in an increase in spiritual well-being. Though this finding seems to be logical, no previous studies are available in this regard. 

One of the most important limitations of this study that can hinder generalizability of the findings was the limited sample size. Therefore, further studies with larger sample size and stratification for socio-economical variables are suggested. 

## Conclusion

Although most of the elderly participating in this study showed a moderate level of spiritual well-being and a low level of happiness, the level of spiritual well-being was approximately higher among the elderly who lived in sanitariums but the level of happiness was higher among the community dwelling elderly adults. It seems that the elderly who lived with their families benefitted from more social and emotional support that affected their level of happiness. On the other hand, it seems that the older adults in sanitariums have more time to think of the meaning of life and its goals. Then, they had a higher level of spiritual well-being. Measures should be taken to increase the older adults’ level of happiness and spiritual well-being. Public education on the importance of happiness and spiritual well-being among the elderly, encouragement of families and specially the aged peoples’ children to increase their emotional and social support of their aged parents, holding programs to help the older adults in recalling their positive memories and thinking about the meaning of life and increasing their attention toward God may also help them feel higher levels of spiritual well-being and happiness. It is also suggested that the nursing students should be taught the concepts of happiness and spiritual well-being (especially among the elderly population). Then, a higher quality of caring may be delivered, especially by nurses who work in sanitariums and the elderly care settings. 
